# Characteristics and Treatment Outcomes of Patients with Tuberculosis Receiving Adjunctive Surgery in Uzbekistan

**DOI:** 10.3390/ijerph18126541

**Published:** 2021-06-17

**Authors:** Anvar Riskiyev, Ana Ciobanu, Arax Hovhannesyan, Kristina Akopyan, Jamshid Gadoev, Nargiza Parpieva

**Affiliations:** 1 Republican Specialized Scientific-Practical Medical Centre of Phthisiology and Pulmonology, 1 Alimov Street, Tashkent City 100086, Uzbekistan; nargizaparpieva@gmail.com; 2 World Health Organization Regional Office for Europe, UN City, Marmorvej 51, DK-2100 Copenhagen, Denmark; aciobanu@who.int (A.C.); hovhannesyana@who.int (A.H.); dr.akopyan@gmail.com (K.A.); 3 Tuberculosis Research and Prevention Centre NGO, Yerevan 0070, Armenia; 4 World Health Organization Country Office in Uzbekistan, 16 Tarobiy Street, Tashkent City 100100, Uzbekistan; gadoevj@who.int

**Keywords:** adjunctive surgical intervention, tuberculosis, Uzbekistan, SORT-IT, operational research

## Abstract

Surgical interventions are performed as an adjunct to pharmacological treatment in Uzbekistan in 10–12% of diagnosed tuberculosis (TB) patients. In this study among patients with respiratory TB who had surgical interventions in Republican Specialized Scientific-Practical Medical Centre of Phthisiology and Pulmonology of Uzbekistan (RSSPMCPP) from January to May 2017, we describe (i) reasons and types of surgical intervention, (ii) post-surgical complications, (iii) histological diagnosis before and after surgery, and (iv) treatment outcomes. There were 101 patients included in the analysis (mean age 36 years; 51% male; 71% lived in rural areas). The main indications for surgical intervention included pulmonary tuberculoma (40%), fibrocavitary, or cavernous pulmonary TB (23%) and massive hemoptysis (20%). Pulmonary resections were the most frequent surgical procedures: segmentectomy (41%), lobectomy or bilobectomy (19%), and combined resection (17%). Ten patients (9%) suffered post-surgery complications. According to histological examination after surgery, TB was confirmed in 81 (80%) patients. For the other 20 patients, the confirmed diagnoses were: lung cancer (*n* = 6), echinococcosis (*n* = 5), post-TB fibrosis (*n* = 5), non-tuberculous pleurisy (*n* = 2), hamartoma (*n* = 1), and pneumonia (*n* = 1). The majority of patients (94%), who underwent surgery, were considered successfully treated. In conclusion, adjunctive surgical therapy can be an option for TB treatment, especially in cases of complicated TB.

## 1. Introduction

Worldwide, tuberculosis (TB) is one of the top 10 causes of death and the leading cause of death from a single infectious agent [1]. Globally, in 2019, an estimated 10 million people developed TB and 1.2 million died from the disease [2].

According to World Health Organization (WHO) data, Uzbekistan remains among the 18 high-priority TB countries in the WHO European Region. Even though its overall TB incidence rate was reversed in 2013 and continues to decline by an average of 6.6% per year, the country’s rising rifampicin-resistant (RR) TB burden ensures that it is in the list of countries with the highest RR-TB burden in the world [1,2]. The latest achievements of the National TB Program regarding RR-TB include detection of 58% of pulmonary RR-TB patients estimated by WHO in 2018 and successful treatment of 57% of RR-TB cases in 2016, this being much lower than the target of 75% for WHO European Region as a whole [2,3]. The main reasons for unfavorable treatment outcomes were death (16%) and lost to follow-up (12%) [2].

Pulmonary and pleural TB may be severe and challenging, even with drug susceptible strains of *Mycobacterium tuberculosis*, and may require a multidisciplinary approach for best management. Moreover, drug-resistant TB and, in particular, multidrug-resistant (MDR) and extensively drug-resistant (XDR) TB frequently occur in patients who have had prior TB episodes and may worsen previously damaged lungs [4,5,6]. TB treatment is complex. Although current WHO recommendations include short treatment regimens for MDR-TB, these are used more often under operational research conditions. Thus, long-term treatment regimens (up to 24 months) are still used.

As new evidence is made available and more is known about drugs and regimens, more patients are surviving [7,8] and it is emerging that other aspects require attention, including adjunctive surgery, emphasized in a comprehensive review of the global tuberculosis network (GTN) based on the consensus of about 100 global experts [5]. As it is known, surgery may play an important role in supporting the diagnosis and treatment of the most complex cases and in improving their therapeutic outcomes. In 2014, the WHO Regional Office for Europe reviewed existing practices and documented expert opinions based on current evidence, which included indications and contraindications for surgical treatment of pulmonary TB and MDR/XDR-TB the conditions for and the timing of surgery, types of surgical intervention, and preoperative and postoperative management [9]. Published literature does not include randomized controlled trials on the efficacy of surgery for the treatment of TB [9]. However, existing research has demonstrated that in countries with a high TB incidence, surgery is still a good option to manage TB complications [10,11] and might be essential as an adjunct to appropriate chemotherapy in MDR-TB patients [12,13,14].

Surgical treatment is performed as an adjunct to TB treatment in Uzbekistan, and during the last five years (2016–2020) it has been used on average in 10–12% of diagnosed TB patients. A systematic assessment has not been undertaken to describe the diagnosis and treatment outcomes of patients who underwent TB surgical adjunctive therapy. We therefore conducted a study, among patients who underwent TB surgical adjunctive therapy, to: (i) describe their sociodemographic and clinical characteristics; (ii) document reasons for and types of surgical intervention and type of post-surgical complications; (iii) compare the histological diagnoses after surgery was performed with the diagnoses made before surgery; (iv) determine treatment outcomes.

Such information will be invaluable for the National TB Program to better understand the role of adjunctive surgery in the overall treatment of TB in Uzbekistan.

## 2. Materials and Methods

### 2.1. Study Design

We carried out a descriptive study involving a review of patient files and treatment cards.

### 2.2. General Setting

Uzbekistan is a lower middle-income country [15] located in the heart of Central Asia with a population of about 33 million, and is administratively divided into 13 regions and the Republic of Karakalpakstan. Tashkent is the capital and the largest city.

### 2.3. National TB Control

Diagnosis and treatment of TB in Uzbekistan are free of charge. TB treatment is provided by Provincial TB hospitals/dispensaries at the provincial level and by TB dispensaries at both the district (for the intensive phase) and the primary health care level (for the continuation phase) for all types of TB patients. The Republican Specialized Scientific-Practical Medical Centre of Phthisiology and Pulmonology (RSSPMCPP) of the Ministry of Health represents the central level, with its main responsibilities being: coordination of TB control activities countrywide; overall oversight of the TB control program, including strategic planning, development, and approval of national policy and guidelines related to TB control; monitoring, evaluation, and surveillance; provision of specialized care to TB patients.

All drug-susceptible, new and previously treated TB patients received the WHO-recommended standardized 6-month duration regimen using first-line TB drugs (FLD). Patients diagnosed with RR-TB were enrolled into treatment using second-line drugs (SLD), which were individualized based on drug susceptibility testing (DST) results. The regimens were designed to include four or more active drugs, and included an injectable (kanamycin/capreomycin), ofloxacin, protionamide, and, depending on the type of resistance detected through drug-resistance testing, the use of ethambutol, pyrazinamide, cycloserine, and para-amino-salicylic acid (PAS). According to country guidelines, an injectable agent was given for a minimum of six months. Typically, all patients with TB are hospitalized during the first two months of the treatment.

The Department of Thoracic Surgery is part of RSSPMCPP and it provides surgical adjunctive treatment for TB. Over 200 surgical interventions for TB treatment are performed in this department annually. The operations performed comply with international standards [16]; the new methods of surgical treatment of pulmonary TB were introduced in 2014 and the indications for surgical treatment in TB patients are based on selection criteria approved by the Ministry of Health [17].

### 2.4. Study Population

All patients diagnosed with respiratory TB who underwent surgical interventions for TB diagnosis and TB treatment in the Department of Thoracic Surgery of RSSPMCPP from 1 January to 31 May 2017 were included in the study.

The patients were selected for surgery based on the decision of the Medical Concilium at RSSPMCPP.

### 2.5. Data Sources and Variables

We used three data sources: (1) medical cards of TB patients at the Thoracic Surgery Department for sociodemographic data (age, sex, place of residence, current tobacco use), clinical data (body mass index (BMI), TB type at the case registration, affected site, TB treatment, bacteriological confirmation, disease localization, presence of cavities, and presence of comorbidities (human immunodeficiency virus (HIV), diabetes mellitus, hepatitis, heart disease, and other comorbidities); (2) logbook of surgery at the Thoracic Surgery Department from which we extracted data on surgical interventions (main indications for surgery, surgically performed procedures, complications after surgery, and results of histological examination before and after surgery); (3) patient ambulatory cards from regional TB centers for collecting data on final TB treatment outcomes. Retrospective data were collected from January to May 2020.

TB treatment outcome, TB type at the case registration and localization of TB disease definitions used in our study were in line with WHO guidelines [18]. We considered cure or treatment completion as a favorable treatment outcome and failure, death or lost to follow-up as an unfavorable outcome. TB patients registered as previously treated included relapse, treatment after failure and treatment after lost to follow-up. Respiratory TB was considered as the localization of disease in the lung parenchyma (pulmonary TB) and pleura (extrapulmonary TB).

### 2.6. Data Analysis

Data were collected using EpiData software (version 3.1, EpiData Association, Odense, Denmark). The final dataset was checked for consistency with original data sources. Analysis was done using Stata software version 15 (Stata Corp, College Station, TX, USA). We described patients’ characteristics, indications for surgical procedures, types of surgical procedures, diagnostic outcomes and treatment outcomes with frequencies and percentages for categorical variables and means (and standard deviation) for continuous variables. Unadjusted relative risks with 95% confidence intervals were calculated to assess the associations between demographic and clinical characteristics and treatment outcomes.

We used RawGraphs web-application to construct an alluvial plot to visualize the change of diagnosis at the pre-surgical and post-surgical stages [19].

## 3. Results

### 3.1. Demographic, Epidemiological, and Clinical Features

There were 101 patients with a diagnosis of respiratory TB who were included in the analysis. All patients had been notified as having TB and all had started on TB treatment before surgery was performed. The mean age of the patients was 36 (SD 13) years. Just over half of the patients were male (51%), most of them lived in a rural area (71%), and over one fourth were smokers (26%). None of the patients were HIV-positive. More than half (58%) were registered as a new patient and the majority (92%) had pulmonary TB. One-fifth (22%) were bacteriologically confirmed before the surgery (the biological specimen was positive by any one of smear microscopy, culture or WHO recommended rapid diagnostics such as Xpert MTB/RIF). Additional clinical and demographic characteristics are presented in Table 1.

### 3.2. Surgery

The main indications for the first surgery were pulmonary tuberculoma (40%), fibrocavitary or cavernous pulmonary TB (24%), and massive hemoptysis (20%) (Table 2).

All 101 TB patients underwent surgical interventions. Seven patients had two surgical interventions and two patients had three surgical interventions. Lung resections of different size using posterolateral thoracotomy were the most frequent surgical procedures. We performed segmentectomy in 41%, lobectomy or bilobectomy in 19%, combined resection in 17%, and pneumonectomy or pleuropneumonectomy in 15% at the first surgery intervention. (Table 3).

The complications after surgeries are shown in Table 4. Ten patients (9%) suffered post-surgery complications after the first intervention, with wound infection being the most frequent complication (*n* = 5). One patient with a wound infection had additionally a residual cavity, bronchial fistula, and bronchopleural thoracic fistula, and a second patient with a wound infection had additionally a residual cavity and pneumothorax.

**Figure 1:**
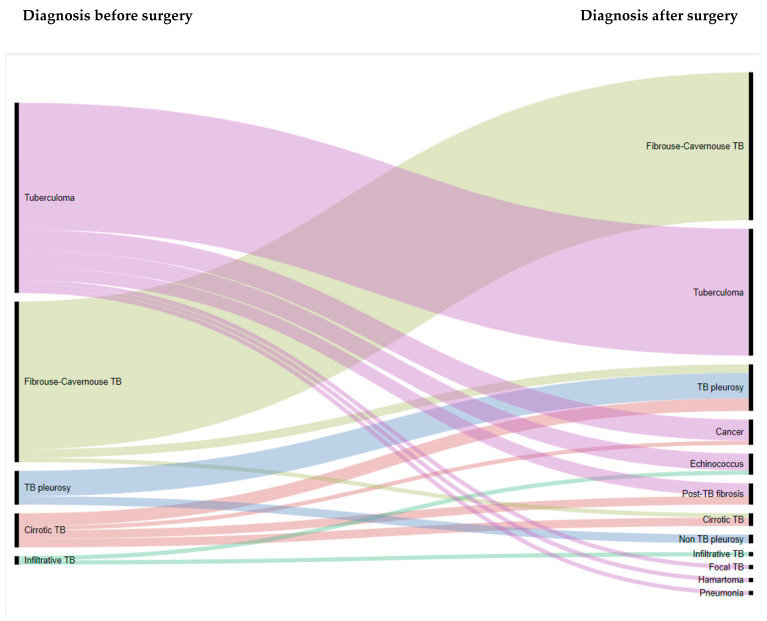
Alluvial plot illustrating the comparison of histological diagnoses after surgery was performed with diagnoses before surgery among patients treated for respiratory tuberculosis who underwent adjunctive surgery at RSSPMCPP in Uzbekistan from January to May 2017 (*n* = 101). RSSPMCPP—Republican Specialized Scientific-Practical Medical Centre of Phthisiology and Pulmonology; TB—tuberculosis.

Based on histological examination of pathological materials removed during the surgery, a TB diagnosis was confirmed in 81 (80%) patients. For the other 20 (20%) patients, the diagnosis was revised as follows: lung cancer (*n* = 6), echinococcosis (*n* = 5), post-TB fibrosis (*n* = 5), non-TB pleurisy (*n* = 2), hamartoma (*n* = 1), and pneumonia (*n* = 1). The comparison of histological diagnoses after surgery with diagnoses before surgery is shown in Figure 1.

### 3.3. Characteristics of Patients with Confirmed TB Post-Surgery

Table 5 shows the clinical characteristics of 81 patients with histological confirmation of TB. Over half of them were new patients (53%) at the case of registration. In the majority of patients, TB disease was localized in the lung parenchyma (86%) and the lesions were unilateral (93%). The presence of cavity before surgery was observed in 43% of TB patients. Most of the TB patients had a bacteriological confirmation after surgery intervention (86%).

### 3.4. Treatment Outcomes of Patients with Confirmed TB Post-Surgery

**Figure 2:**
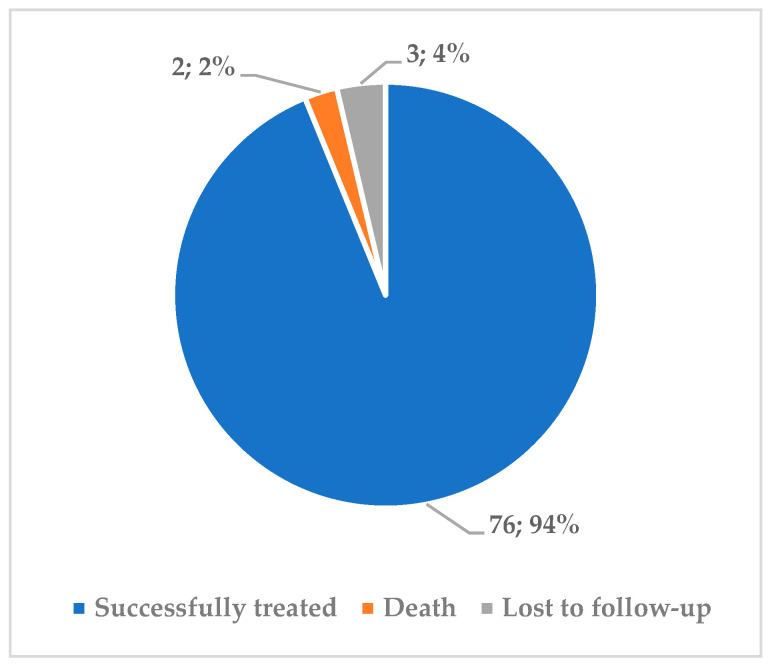
Treatment outcomes among 81 patients with respiratory tuberculosis who underwent adjunctive surgery at RSSPMCPP in Uzbekistan from January to May 2017. RSSPMCPP—Republican Specialized Scientific-Practical Medical Centre of Phthisiology and Pulmonology.

Most of the 81 TB patients who underwent surgery were considered successfully treated according to WHO’s definitions (94%, Figure 2). Due to small sample sizes and skewed distribution of categories of patients that included clinical and demographic characteristics, an analysis of predictors of treatment outcome was hampered because of insufficient power. Data are not shown.

## 4. Discussion

Historically, before the development of anti-TB drugs, surgery was the only treatment available for TB [9]. With the introduction of modern anti-TB chemotherapy, surgery was largely abandoned [20] and, until the present day, chemotherapy has been the main treatment modality for TB, including its drug-resistant forms. According to literature reviews, in industrialized countries the number of surgical interventions fell considerably because the incidence and prevalence of TB declined, the anti-TB medicines were effective and the indications for surgery became further limited. It is only in the countries of the former Soviet Union that surgery has remained a relatively widespread option for the treatment of TB [9,20]. Moreover, the national TB guidelines of some countries of the former Soviet Union recommend that persistence of cavitary lesions on a chest X-ray is one of the main indications for surgical treatment of TB [11,17]. In addition, surgery is now increasingly used to treat complications of pulmonary TB, particularly in patients with drug-resistant TB who do not respond to medical treatment.

In our study we described surgical intervention as an adjunctive therapy for treating TB in Uzbekistan. Our study findings can be compared with other studies.

In our study we observed that the main indications for surgical interventions were pulmonary tuberculoma, fibrocavitary or cavernous pulmonary disease, and massive hemoptysis. The rates of fibrocavitary or cavernous pulmonary TB associated with hemoptysis in countries of the former Soviet Union with a high TB burden can be explained by characteristic late referrals of patients with TB (all forms) for medical attention. This results in a large number of patients with advanced, extensive cavernous disease with strongly expressed and irreversible morphological changes in the lungs, and these respond poorly to standard anti-TB treatment [11].

It is estimated that 5–14% of patients presenting with hemoptysis will have life-threatening hemoptysis known as massive hemoptysis [21,22]. A study conducted in Shanghai, China showed that surgical treatments provided superior results in the management of massive hemoptysis [23]. Previously published Russian studies mention that early surgical treatment of tuberculoma led to better patient recovery and a reduction in the amount of chemotherapy treatment [24,25]. Persisting tuberculomas, larger than two centimeters, in a sputum smear/culture negative patient is usually treated with extended anti-TB medication, but others argue that the inflammatory barrier is an obstacle to obtaining adequate drug concentrations [26]. A study conducted in Georgia also stated that the presence of lesions (cavities, tuberculomas, and empyema) were the main indications for surgical interventions [11].

According to the published literature, the principal types of surgical intervention used to treat TB are lung resections of different sizes using posterolateral thoracotomy [13,27,28], similar to what we observed in Uzbekistan. In Kazakhstan, the Russian Federation and Ukraine, thoracoplasty is also still widely used when lung resection is contraindicated [29,30].

In our study, post-operative complications from surgical interventions in TB patients were few in number, and this compares favorably with the 9–26% complication rate that has been reported in the scientific literature [27,28,31].

Our study highlighted the importance of histological examination. For one in five of our patients, the diagnosis of TB was not confirmed. In many patients that present as tuberculoma, a differential diagnosis should be done and the diagnosis carefully considered. Biopsy and histopathology are considered the gold standard in diagnosing tuberculomas as imaging studies are often misleading in the diagnosis of this rare condition [32].

Our study highlighted a high treatment success amongst TB patients undergoing surgical resection. These findings are in line with a systematic review and meta-analysis of adjunctive pulmonary resection for TB patients with the inclusion of 15 studies (a total sample size of 949), which showed the overall cure rate as high as 84% [33]. A South Korean study addressing surgical intervention for MDR/XDR-TB in 72 patients between 1996 and 2008 included 26 patients with XDR-TB and reported 90% favorable outcome following surgical resection [31].

Our study has some limitations. First, the current study was observational. Second, TB patients were included in the study over a short period of time. Third, we were unable to compare the characteristics of similar groups of patients undergoing surgery compared with those not undergoing surgery. Finally, our intention to analyze baseline demographic and clinical characteristics in relation to treatment outcomes was not possible because of insufficient sample size and skewed distribution of variables.

## 5. Conclusions

In conclusion, as our study and other published studies are observational it is not possible to make clear evidence-based recommendations regarding the role of surgery in the treatment of respiratory tuberculosis. Our findings show that in many cases of complicated TB with localized lesions and tissue destruction, adjunctive surgical therapy can play a major role in improving clinical outcome.

In this context, additional research is necessary in order to make evidence-based recommendations and guidelines regarding the role of surgery in the treatment of respiratory TB.

## Figures and Tables

**Table 1:** Demographic, epidemiological, and clinical features of patients diagnosed with respiratory tuberculosis who underwent adjunctive surgery at RSSPMCPP in Uzbekistan from January to May 2017 (*n* = 101).

Characteristics	*n* (%)
**Age groups**	
≤35 years	55 (54.5)
˃35 years	46 (45.5)
**Sex**	
Male	51 (50.5)
Female	50 (49.5)
**Place of residence**	
Urban	29 (28.7)
Rural	72 (71.3)
Current smoker *	
Yes	26 (26.3)
No	73 (73.7)
**HIV status**	
Positive	0 (0.0)
Negative	101 (100.0)
**Diabetes mellitus**	
Yes	7 (6.9)
No	94 (93.1)
**Hepatitis**	
Hepatitis B	7 (6.9)
Hepatitis C	7 (6.9)
Both hepatitis B and C	2 (2.0)
None	85 (84.2)
**Heart disease**	
Yes	16 (15.8)
No	85 (84.2)
**Other co-morbidities**	
Yes	8 (7.9)
No	93 (92.1)
**BMI (mean = 23.9, SD 3.1)**	
<18.5 kg/m^2^	5 (5.0)
18.5–24.9 kg/m^2^	56 (55.5)
≥25.0 kg/m^2^	40 (39.5)
**TB type**	
New	59 (58.4)
Previously treated	42 (41.6)
**TB treatment before surgery**	
FLD	68 (67.3)
SLD	33 (32.7)
**Localization of TB disease before surgery**	
Lung parenchyma (pulmonary)	93 (92.1)
Pleural (only)	8 (7.9)
**Affected side before surgery**	
Unilateral left	46 (45.5)
Unilateral right	48 (47.5)
Bilateral	7 (6.9)
**Presence of cavity before surgery**	
Yes	38 (37.6)
No	63 (62.4)
**Bacteriological confirmation before surgery**	
Yes	22 (21.8)
No	79 (78.2)

BMI—body mass index; HIV—human immunodeficiency virus; TB—tuberculosis; FLD—first line drugs; SLD—second line drugs. RSSPMCPP—Republican Specialized Scientific-Practical Medical Centre of Phthisiology and Pulmonology; * Missing data was excluded: current status of smoking (*n* = 2).

**Table 2:** Main indications for the first surgical procedures in patients with respiratory tuberculosis who underwent adjunctive surgery at RSSPMCPP in Uzbekistan from January to May 2017 (*n* = 101).

Main Indication of First Surgery	*n* (%)
Pulmonary tuberculoma	40 (39.6)
Fibrocavitary or cavernous pulmonary	24 (23.8)
Massive hemoptysis	20 (19.8)
Pachypleuritis	8 (7.9)
Empyema	3 (3.0)
Cirrhotic pulmonary TB	2 (2.0)
Atelectasis	1 (1.0)
Bronchial fistula	1 (1.0)
Irreversible TB progression	1 (1.0)
Caseous pneumonia	1 (1.0)

RSSPMCPP—Republican Specialized Scientific-Practical Medical Centre of Phthisiology and Pulmonology; TB—tuberculosis.

**Table 3:** Surgical procedures performed on patients with respiratory tuberculosis at RSSPMCPP in Uzbekistan from January to May 2017 (*n* = 101).

Type of the Surgery	1st Surgery (*n* = 101)	2nd Surgery (*n* = 7)	3rd Surgery (*n* = 2)
	*n* (%)	*n* (%)	*n* (%)
Segmentectomy	41 (40.6)	2 (28.6)	0 (0.0)
Lobectomy and bilobectomy	19 (18.8)	0 (0.0)	0 (0.0)
Combined resection	17 (16.8)	1 (14.3)	0 (0.0)
Pneumo- or pleuropneumonectomy	15 (14.9)	0 (0.0)	0 (0.0)
Thoracoplasty, pleurectomy, decortication	5 (5.0)	0 (0.0)	0 (0.0)
Bronchial occlusion	2 (2.0)	1 (14.3)	0 (0.0)
Wedge resection	1 (1.0)	0 (0.0)	0 (0.0)
Bronchial reamputation	1 (1.0)	0 (0.0)	0 (0.0)
Extra pleural thoracoplasty	0 (0.0)	2 (28.6)	1 (50.0)
Thoracentesis and thoracostomy	0 (0.0)	1 (14.3)	1 (50.0)

RSSPMCPP—Republican Specialized Scientific-Practical Medical Centre of Phthisiology and Pulmonology.

**Table 4:** Complications of surgery performed in patients with respiratory tuberculosis at RSSPMCPP in Uzbekistan from January to May 2017.

Description of the Complication	1st Surgery (*n* = 101)	2nd Surgery (*n* = 7)	3rd Surgery (*n* = 2)
	*n* (%)	*n* (%)	*n* (%)
Residual cavity	2 (2.0)	1 (14.3)	0 (0.0)
Bronchial fistula	2 (2.0)	0 (0.0)	0 (0.0)
Bronchopleural thoracic fistula	1 (1.0)	0 (0.0)	0 (0.0)
Wound infection	5 (5.0)	1 (14.3)	0 (0.0)
Pneumothorax	1 (1.0)	0 (0.0)	0 (0.0)
Hemothorax	0 (0.0)	1 (14.3)	0 (0.0)
Other complications	4 (4.0)	1 (14.3)	0 (0.0)
**Total number of complications**	**15**	**4**	**0**

RSSPMCPP—Republican Specialized Scientific-Practical Medical Centre of Phthisiology and Pulmonology.

**Table 5:** Clinical characteristics of patients with histological confirmed active tuberculosis after surgery was performed at RSSPMCPP in Uzbekistan from January to May 2017 (*n* = 81).

Characteristics	*n* (%)
**TB type at the case registration**	
New	43 (53.1)
Retreatment	38 (46.9)
**Localization of TB disease**	
Lung parenchyma (pulmonary)	70 (86.4)
Pleural (only)	11 (13.6)
**Affected side**	
Unilateral left	40 (49.4)
Unilateral right	35 (43.2)
Bilateral	6 (7.4)
**Presence of cavity before surgery**	
Yes	35 (43.2)
No	46 (56.8)
**Bacteriological confirmation after surgery**	
Yes	70 (86.4)
No	11 (13.6)

RSSPMCPP—Republican Specialized Scientific-Practical Medical Centre of Phthisiology and Pulmonology, TB—tuberculosis.

## Data Availability

The data that support the findings of this study are available from the corresponding author upon reasonable request.
